# Degraded image enhancement by image dehazing and Directional Filter Banks using Depth Image based Rendering for future free-view 3D-TV

**DOI:** 10.1371/journal.pone.0217246

**Published:** 2019-05-23

**Authors:** Imran Uddin Afridi, Tariq Bashir, Hasan Ali Khattak, Tariq Mahmood Khan, Muhammad Imran

**Affiliations:** 1 Department of Computer Science, COMSATS University Islamabad, Pakistan; 2 Department of Electrical Engineering, COMSATS University Islamabad, Pakistan; Huazhong University of Science and Technology, CHINA

## Abstract

DIBR-3D technology has evolved over the past few years with the demands of consumers increasing in recent times for future free-view 3D videos on their home televisions. The main issue in 3D technology is the lack of 3D content available to watch using the traditional TV systems. Although, some sophisticated devices like stereoscopic cameras have been used to fill the gap between the 3D content demand and 3D content supply. But the content generated through these sophisticated devices can not be displayed on the traditional TV systems, so there needs to be some mechanism which is inline with the traditional TV. Furthermore, the huge collection of existing 2D content should be converted to 3D using depth image-based rendering techniques. This conversion technique can highly contribute in overcoming the shortage problem of the 3D content. This paper presents a novel approach for converting 2D degraded image for DIBR 3D-TV view. This degraded or noisy/blur image is enhanced through image dehazing and Directional Filter Bank (DFB). This enhancement is necessary because of the occlusion effect or hole filling problem that occurs due to imperfect depth map. The enhanced image is then segmented into the foreground image and the background image. After the segmentation, the depth map is generated using image profiles. Moreover, Stereoscopic images are finally produced using the DIBR procedure which is based on the 2D input image and the corresponding depth map. We have verified the results of the proposed approach by comparing the results with the existing state-of-the-art techniques.

## Introduction

Depth Image Based Rendering three-dimensional Television (DIBR-3DTV) technology added a new dimension to the world of entertainment. The advancement in DIBR-3D changed the conventional two-dimensional (2D) entertainment media to more realistic one. This diverse technology has adopted by many entertainment platforms such as TV, cinemas and video gaming [1]. The 2D video games have been converted to 3D games by using Kinect camera [1] in XBOX [2], so people can enjoy playing games in a more realistic virtual world representation. Film industries are gaining enormous financial benefits by introducing DIBR-3D technology and their income increased exponentially e.g. ‘avatar’, a DIBR-3D enabled movie released in 2009 and it became the game changer in the film industry which earned tenfold of its investment [3]. Multiple broadcasting service providers use DIBR-3D technology and many European and Asian countries have commenced its transmission. DIBR-3D TV is the revolution in traditional television systems and made it even more smart where viewers enjoy lifelike scenes on their traditional home television [4].

DIBR-3D TV contents were first introduced in Advance Three-Dimensional Television System Technology (ATTEST) [4] by Fehn *et. al*. [5]. Information Society Technology (IST) initiated the ATTEST project in March 2009 with the support of European commission. ATTEST was a 2 years project in which number of industrial and academic partners worked together. The goal of this collaboration was to build up a pliable, 2D suitable and commercially viable 3D-TV for broadcasting system environments.

A novel approach based on combined transmission of the depth map and corresponding 2D video is proposed by Fehn *et. al*. [5]. Depth map is the gray-scale image, consist of information related to the distance of objects in the scene from the viewpoint. The depth map and the corresponding input 2D video/image are combined to generate the left and right view using DIBR procedure for the DIBR-3D TV system [4]. 3D technology has improved over the past few years and the generation of 3D contents has been enhanced significantly. 3D contents for DIBR-3D TV can be generated by three different ways.

1. Direct 3D content generation.
2. Combined transmission of 2D data and corresponding depth map.
3. 3D content generation from a 2D image.

In first, the direct 3D content generation requires multiple expensive special high resolution cameras. This mechanism is not an economical and feasible solution in terms of complex coding [6], high bandwidth requirement [7] and data synchronization issues at the receiving side.In second, the 2D data and the corresponding depth map information are transmitted together. At the receiving end, the synthesized 3D image is generated using depth map information of 2D data [4]. In third, the generation of 3D contents from a 2D image is a cumbersome procedure due to the absence of information required to obtain the 3D scene. Although 2D scene possesses fewer cues for 3D view generation, still researchers have managed to acquire efficient results by using the per pixel depth information of the 2D image.

The generation of 3D scene from a 2D image requires two steps. In the first step, depth map (per pixel depth information) is extracted from 2D data. In the second step, the obtained depth map image is combined with the original 2D image using DIBR. Above all, extracting efficient depth information from a 2D image plays an important role in 3D view generation. Numerous methodologies have been proposed to produce depth map [8–11].

In [12], defocus depth map is calculated using degraded or defocus 2D image. This defocus depth map information can introduce occlusion effect in the synthesis 3D images if the quality of the depth map is not upto mark. The occlusion effect compromise the quality of the 3D contents since the occluded area can be seen while viewing the 3D contents. To tackle the occlusion effect. Wang *et al*. [13] proposed depth map enhancement methodology by deploying three different types of constraints on reference and target patches in depth map. The occlusion problem is addressed in single as well as in multiple view using global optimization method [14]. Here efforts have been made to decrease occlusion effect by improving the quality of depth map using different operations on depth map. Although, quality of the depth map can be improved and occlusion would be decreased if we enhance the quality of the corresponding input image. One of the main contributions of this paper is to minimize the occlusion problem, that is mainly occurs due to the imperfection of depth-map. This occlusion effect or hole filling issues increase due to the degradation of input image. This work mainly emphasize on the enhancement of degraded image using dehazing process and trough directional filter banks. After enhancement, depth hypothesis are applied to generate the depth-map. At the end, DIBR system is used to generate the left and right images to further calculate the occlusion effect. The synthesized images are used to create the final anaglyph image for the end users. Though this approach has been used in state of the art, but we present Directional Filter Bank Depth Image based Rendering System (DFB-DIBR) which improves the Peak Signal to Noise Ratio (PSNR), Structure Similarity Index Measure (SSIM) and Universal Quality Index (UQI) of the depth map. The enhanced depth map would decrease occlusion effect which ultimately generates good quality 3D view.

Rest of the paper is organized as follows: Section 1 introduces the related work through state of the art literature review in 3D technology related to DIBR. Section 2 presents the proposed system and its working methodology for generation of depth map and 3D content generation. Experiment and Results have been described in Section 3 while Paper has been concluded in Section 4.

## 1 Related work

The existing depth map generation algorithms are mainly classified in two categories: Automatic method and Semi-Automatic method. In Automatic method, different depth cues are considered such as focus and defocus information [9, 10] where image’s focus data is considered by varying the focus parameters of a camera. Yang *et al*. [8] proposed a method which classify the input image into the three categories i.e. landscape, closeup and the linear perspective image. After classification, each class’s depth map is produced. In [12], defocus / blur information is calculated at the edges of objects to approximate the defocus depth map. Huang *et al*. [11] proposed an algorithm for estimation of depth map which is produced by finding defocus/blur edge information using wavelet transformation and the canny edge detector. Depth from objects motion in video frames has been proposed by [15]. Tsai *et al*. [16] proposed Gaussian mixture model (GMM) and (SLIC) super pixel simple linear iterative clustering algorithm to generate the initial depth map. The initial depth map further refined using edge’s information and various scanning path mode. In [17], the formation of the depth map is based on Sum of Absolute Difference (SAD) of neighborhood pixels of two same images. Williem *et al*. [18] proposed anaglyph image based approach to generate the depth map. The obtained depth map assists the algorithm to colorize the synthesis images. The defocus depth map estimation in [19] has achieved in two phases. In the first phase, the defocus/blur image is re-blurred. In the second phase, the ratio of edges’ information is taken of the re-blurred and the input image. Although, automatic methods of depth map generation is less computational expensive, still these methods compromise on the quality of depth map. All the above depth map generation methods are relying on priory defined geometrical information.

On the contrary, the depth map estimation problem is solved by Deep Convolutional Neural Network (DCNN) without pre-defined image’s structure information. The pioneer work on depth map estimation from an image is attributed to [20], in which CNN model is trained jointly with Conditional Random Field (CRF) to learn the continuous nature of the depth map image’s structure. Luo *et al*. [21], proposed dual neural network architecture. The view synthesis network is used to produce the right view of the input image. The stereo matching network uses the right synthesis view and the input image to produce the depth map. In [22], unsupervised DCNN architecture is proposed. The network predict probabilistic depth map and combine it with the input to generate the side by side or anaglyph 3D view. A semi supervised deep network is proposed in [23]. In [23], the network architecture takes benefits of supervised as well as unsupervised learning techniques. The network uses 3D laser to captured ground truth data for supervised learning. On the other hand, network acquires stereo matching geometry using stereo cameras to predict the depth map in unsupervised way. Depth map from out of focus image is generated using deep learning in two phases [24], In first phase, network takes an out of focus image and generates the defocus depth map. In second phase, the depth map is used by network to refocus the out of focus image. Hand crafted features play an important role to learn the best feature of the given data using deep neural networks. Hand crafted and deep network features have been used synergistically to estimate the defocus depth map from an input defocus image [25]. The architecture uses the advantages of hand crafted and deep network features to overcome the weakness of each other. All the deep learning architectures are fine-tuned to learn the best features of the training data. Hand crafted features can assist the deep network to learn the reliable features of the training data. Our proposed algorithm can be an efficient preprocessing step of the deep network to learn the best features of the given data.

The efficient 3D view depends on high quality depth map generated from an image. To have promising 3D results, semi-automatic methodologies have been proposed with little users’ interference. Generation of depth map based on local depth hypothesis is proposed by [26]. In [26], depth map is produced using vanishing point which are considered farthest points in an input image. The dark shades of the gray scale image are assigned to these farthest points and bright shades to closest points. Phan *et al*. [27] proposed integrated algorithms of scale space random walk and graph cut segmentation to generate the depth map. In [28], a semi-automatic method is proposed to generate the depth map. The system takes random scribbles from the users which denote the far and nearest points in the image. These scribbles generate the initial sparse depth map. The welsch M-estimator is used to convert the sparse depth map to dense depth map. Though, semi-automatic methods can produce the good quality depth map for the 3D scene. However, such methods are time consuming and incompatible to real time applications.

In recent times, an extended automatic method has made appearance. In such mechanism, machine learning techniques have been used to train a huge repository of (RGB+Depth) which contained consistent depth map images of queried images. The working rule of the system is based on the structure similarity of the images. The trained (RGB+Depth) repository has been used in [29–31]. In [29], kNN (k nearest neighbors) algorithm is used to search relative images and the respective depth map images of the queried image. By fetching multiple similar images with the depth map images, only the structurally similar image + depth are selected and the rest of it is removed. Herrera *et al*. [30] proposed Local Binary Pattern (LBP) based features to retrieve similar images from the (RGB+Depth) repository. Then, the corresponding depths are combined using the correlation weighting scheme. In [31], a huge synthetic (RGB + depth) database of soccer game is created. The algorithm transfers gradients of images’ depth information from synthetic dataset and find the refined depth map using spatio-temporal methodology.

Though, (RGB+Depth) based systems perform well in the respective domain. However, such systems require huge data sets of color images along its depth map images. It also required system training and exhaustive search to find the corresponding results. The main objective of researchers is to provide the view of hyper-reality on traditional TV systems. Researchers are achieving this milestone through constant prodigious efforts. The constant nagging on the development and betterment of hyper-realism on traditional screens make researchers more efficient and efficacious to achieve the required results of 3D contents. The perpetual desire of consumers makes researchers eager to accomplish the goal of advancement in 3D technology. In order to transmit 3DTV data efficiently, transmission requires data compression [32]. This compression creates artifacts at the receiving side i.e. data become blur/noisy. This degraded image data caused occlusion effects which compromise the quality of the 3D view. To address the occlusion effect in single and multi degraded view, the data needs to be enhanced. In [33], degraded data is enhanced using Discrete Cosine Transform (DCT) by considering all the three attributes (brightness, Contrast, Color) of the RGB image. Yang *et al*. [34] proposed a multi-lateral guided filter to enhanced the degraded depth map. The system creates the Macro Super Pixel data structure where the priors of depth and color are used as reference to guide the Gaussian kernels. We are proposing a novel approach to convert a degraded (noisy/blur) image to a quality 3D scene. In the proposed system, the conversion from a degraded image to 3D scene required to enhance the degraded image using the dehazing procedure and DFB [35]. The DBF has many applications such as fingerprint enhancement, image denoising, edges detection etc. The enhanced image is segmented using k-mean classify algorithm. The enhanced information ultimately creates the effective depth map which becomes the concrete foundation for the efficient 3D content generation. After the segmentation, the depth map is generated using image profiles. The depth map is further refined by bilateral filter and then converted to stereoscopic images using DIBR [36]. At the end, the synthesized left and right images are combined to produce 3D view. The proposed calculation is efficient as far as computational and memory confinements. The tested data sets are available online [37] [38] [39]. We have complied with the term of service for the websites from which we have collected data.

## 2 Directional Filter Bank Depth Image based Rendering System (DFB-DIBR)

**Fig 1:**
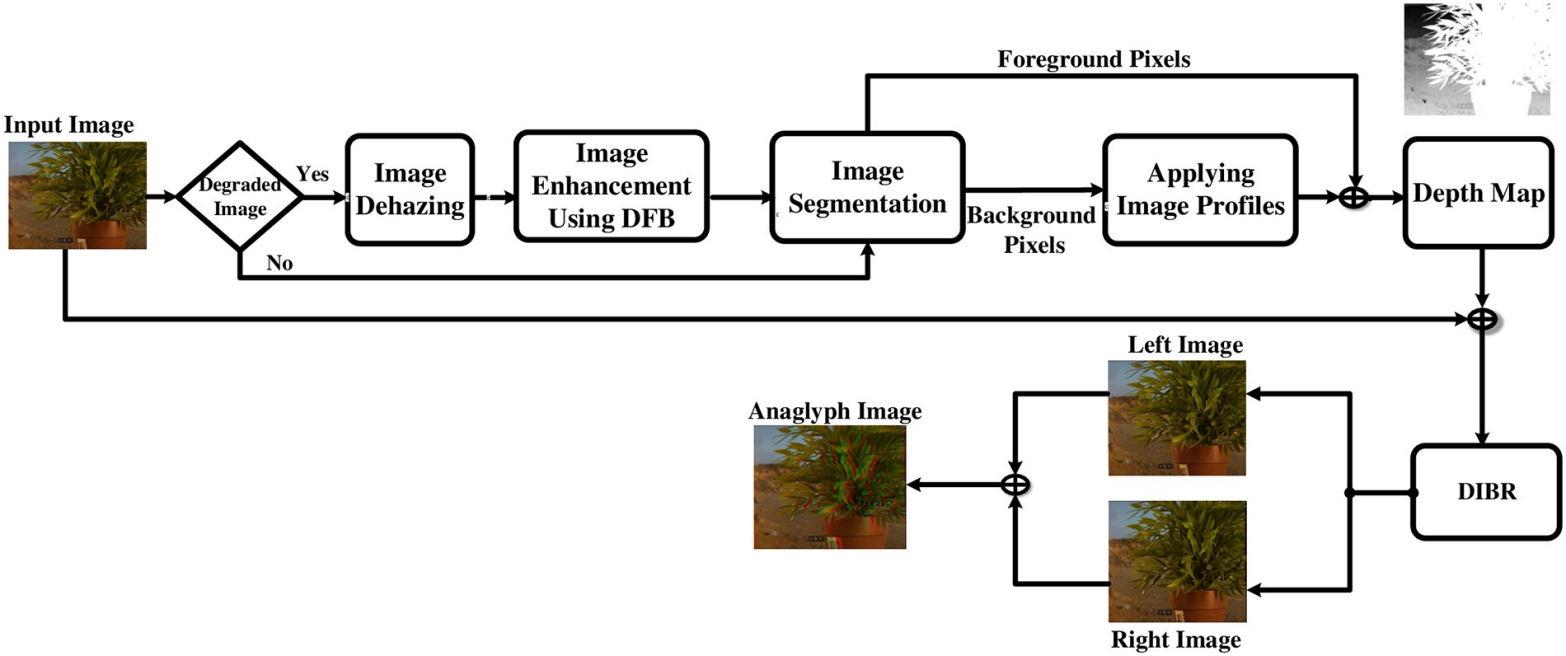
Flow for the proposed Directional Filter Bank Depth Image based Rendering System (DFB-DIBR).

A novel approach to generate 3D view is proposed in this paper. Fig 1 is the block diagram of the purposed system. The DFB-DIBR consists of following parts: Image Dehazing, Noisy/Blur Image Enhancement using DFB, grouping background pixels of similar intensity using k-mean, Applying image profiles or depth hypothesis on background pixels, depth map generation and refinement, creating synthesized left and right image using DIBR, creating Anaglyph image or 3D view. It is shown in Fig 1. A blur/noisy image is inserted to the system. The image is dehazed first. Then the dehazed image is enhanced using DBF. After enhancement, the segmented foreground (white) and background (black) image is inserted. The foreground is a bright region and does not contain any depth discontinuity. On the other hand, the background region has depth variation and we are assuming that pixels of similar intensity have similar depth. We have used K-means classification algorithm to group similar intensity pixels and assigned the same depth value at the next stage. Image profile/depth hypothesis is assigned to the classified background which combined with the foreground pixels to generate the initial depth map. The initial depth map is further cultured to retrieve the refined depth map. In next turn, the refined depth map is integrated with the input image to generate synthesized stereoscopic images using DIBR. At the final stage, the synthesized images are combined to generate 3D virtual view. Each part of the system is described in the following parts.

### 2.1 Image dehazing

**Fig 2:**
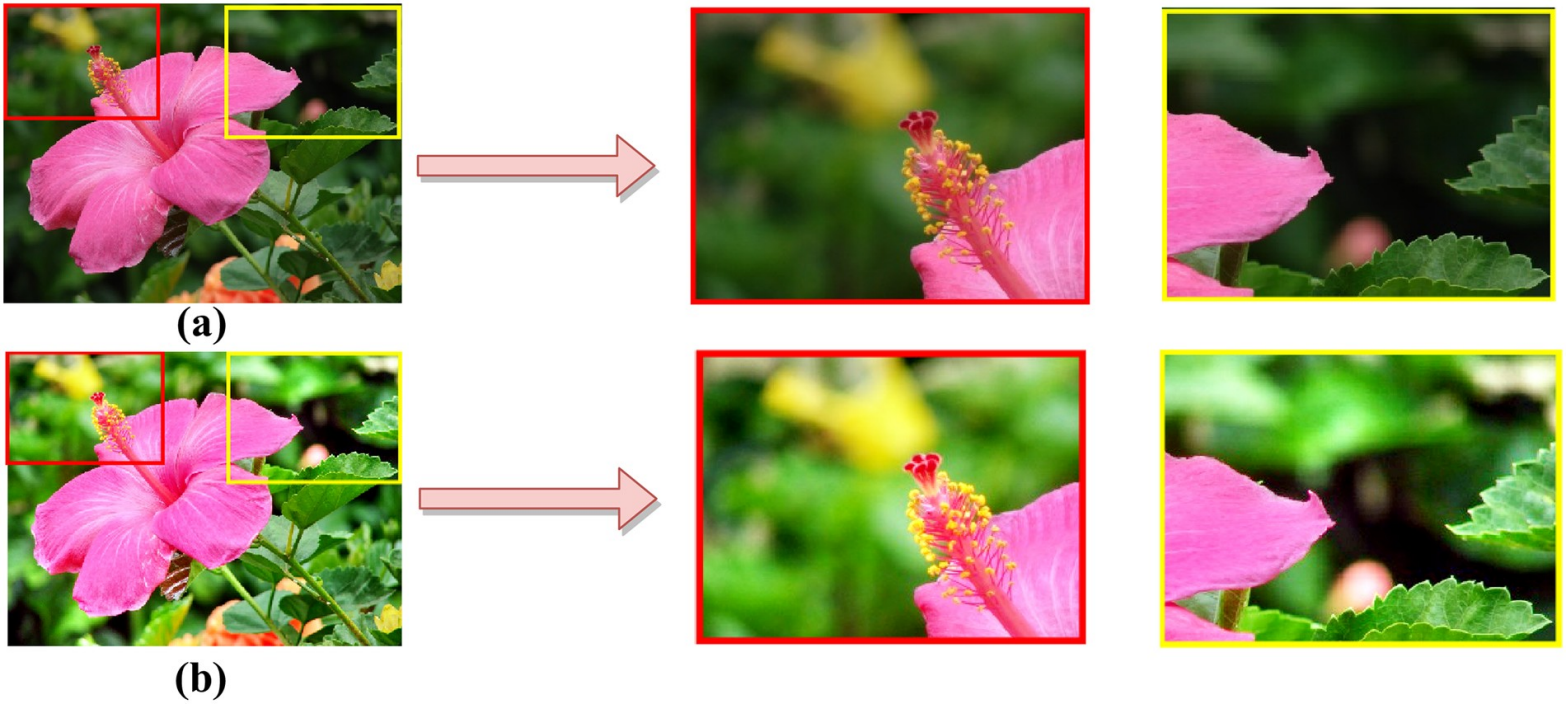
(a) Original image (b) Dehazed image.

The intensity variations between foreground and background can cause non-uniform illumination. The region with varied contrast can be modeled as haze with less visibility. For greater clarity of the objects presents in the background region, we resort to using a de-hazing algorithm, put forward in [40] for improving the visibility of images captured in outdoor scenarios. This is a non-local method that removes haze from an image. Our objective here is to restore the true color and contrast of objects that are usually present in less visible region as shown in Fig 2. A hazy image is a complex combination of true scene, usually present in foreground region, and global error (non-uniform illumination). This phenomenon fits well into the haze model. It has been observed that a haze-free image in the RGB domain can be represented well with few-hundred distinct colors, that form spherical clusters in RGB space. The presence of haze in pictures modify the clusters into straight-line shape, referred to as haze-lines. Using that haze-line, a regularized inverse algorithm presented to make the image haze-free. We adopted this inverse process for its contrast-improvement behavior and efficient computational structure nature that is linear in size of the image. Fig 2 shows the original and the dehazed image.

### 2.2 Blur/Noisy image enhancement using Directional Filter Bank

**Fig 3:**
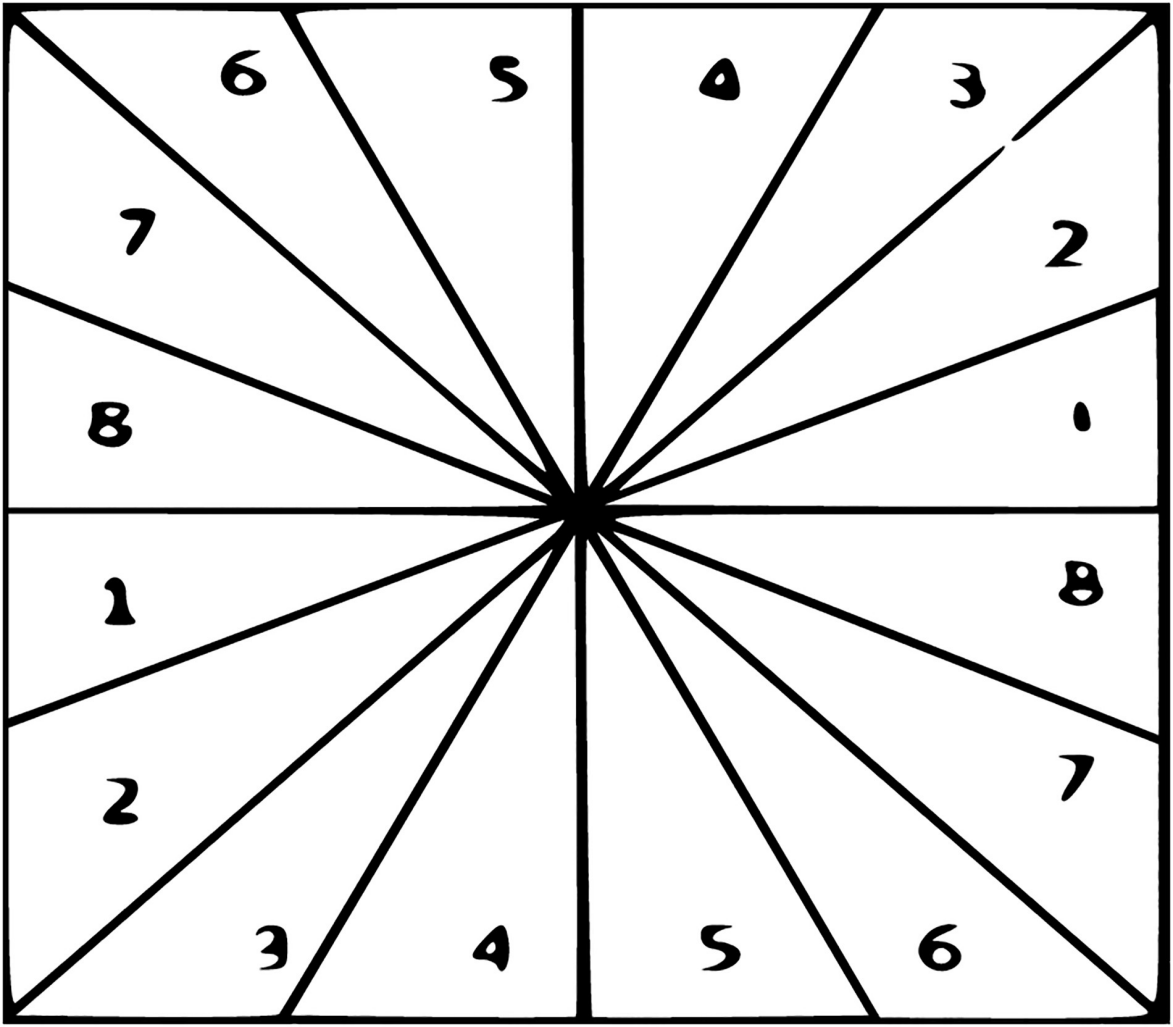
2D frequency partitioning.

Directional filter bank (DFB) was initially proposed by [35]. DFB has been used in many image processing applications such as fingerprint enhancement [41], edges detection [42], image denoising [43] etc. DFB has the uniqueness to disintegrate the multidimensional signal in to few directional sub-band. The DFB can detect and present signal eccentricity in the form of edges lying on the blur/noisy surfaces. The DFB is carry out by an n-layers tree-structure. Due to the n-layers tree-structure, the signal can be decompose in to 2^*m*^ sub-bands with wedge-shape frequency partitioning as shown in Fig 3.

At each decomposition level, the DFB permit for various number of directions. The DFB is also capable of detecting directionality of the coefficient at the high frequency. The Coarse acquisition is provided by low pass sub-bands and directional information is provided by high pass sub-bands. The edges can appear in an image at any range and direction. It is important to acquire the reaction of an edge filter at any self-assertive position and coordinates. DFB is an essential transform that offers the idealize reproduction i.e. the initial signal can be precisely reproduced from its exterminating mediums. The *F*_0_(*ω*) and *F*_1_(*ω*) represent the low pass and high pass filter responses. The Wedge shaped frequency responses are acquired by applying the Checkerboard filter. The wedge responses are helpful for capturing the edges at different scales which result in effective edge detection. *F*_1_(*ω*) provides edge information and a Checker board filter is illustrated by Eq 1.

$$k\left(n{}_{1},n{}_{2}\right)=1/2\left[F{}_{0}\left(-n{}_{1}^{2}\right)F{}_{0}\left(-n{}_{2}^{2}\right)-n{}_{1}n{}_{2}F{}_{0}\left(-n{}_{1}^{2}\right)F{}_{0}\left(-n{}_{1}^{2}\right)\right]$$ (1)

Moreover *F*_0_(*ω*) and *F*_1_(*ω*) fulfill Eq 2.

$$|F{}_{0}{\left(\omega \right)}^{2}+F{}_{1}{\left(\omega \right)}^{2}|=1$$ (2)

Transfer function *T*_*f*_ (*n*1, *n*2) that is achieved from the checkerboard filter. It is given in Eq 3

$$T{}_{f}\left(n{}_{1},n{}_{2}\right)=1/2[F{}_{0}\left(-n{}_{1}n{}_{2}\right)F{}_{0}\left(-n{}_{1}^{-1}n{}_{2}\right)-n{}_{2}F{}_{1}\left(-n{}_{1}n{}_{2}\right)F{}_{1}\left(-n{}_{1}^{-1}n{}_{2}\right)$$ (3)

Eq 4 shows the response of the ideal fan filter.

$$T{}_{f}\left(e^{j\omega 1},e^{j\omega 2}\right)=k\left(e^{i(\omega 1+\omega 2)/2},e^{i(\omega 1+\omega 2)/2}\right)$$ (4)

*F*_1_*ω* obtained the high frequency components that is useful in capturing edge information. The directional derivative of a two-dimensional function is represented by D(x,y). Eqs 5 and 6 show directional derivatives of the function at different orientations.

$$D{}_{1}^{0}\left(r,\theta \right)=cos\left(\theta \right)D\left(x,y\right)$$ (5)

$$D{}_{1}^{\pi /2}\left(r,\theta \right)=sin\left(\theta \right)D\left(x,y\right)$$ (6)

The order of the derivative is expressed by subscript and the angle of the derivative direction is indicated by superscript. It is obvious that the function D1 can incorporate at an arbitrary orientation ‘*ϕ*’ using Eq 7 and can be an edge filter.

$$D{}_{1}^{\phi }\left(r,\theta \right)=cos\left(\phi \right)D{}_{1}^{0}\left(r,\theta \right)+sin\left(\phi \right)D{}_{1}^{\pi /2}\left(r,\theta \right)$$ (7)

*D*_1_ being a directional derivative can be developed at a random orientation as a linear combination of basis filter $D{}_{1}^{0}$ and $D{}_{1}^{\pi /2}$, *cosϕ* and *sinϕ* are assigned as interpolation functions. The function D(x,y) is undeviating and used as a 2D directional filter. This filter is further useful in extracting image’ edges at various orientation and results in precise edge response. Edges consist of directional information where as the noise does not have directional information.

**Fig 4:**
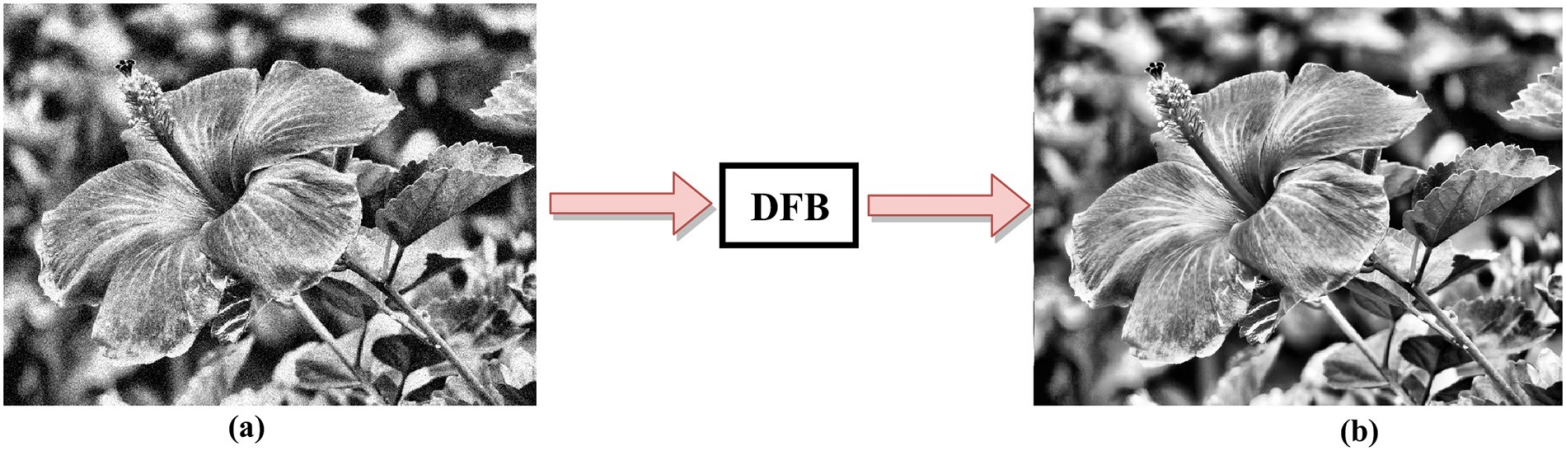
(a) Noisy image (b) Noise free image.

**Fig 5:**
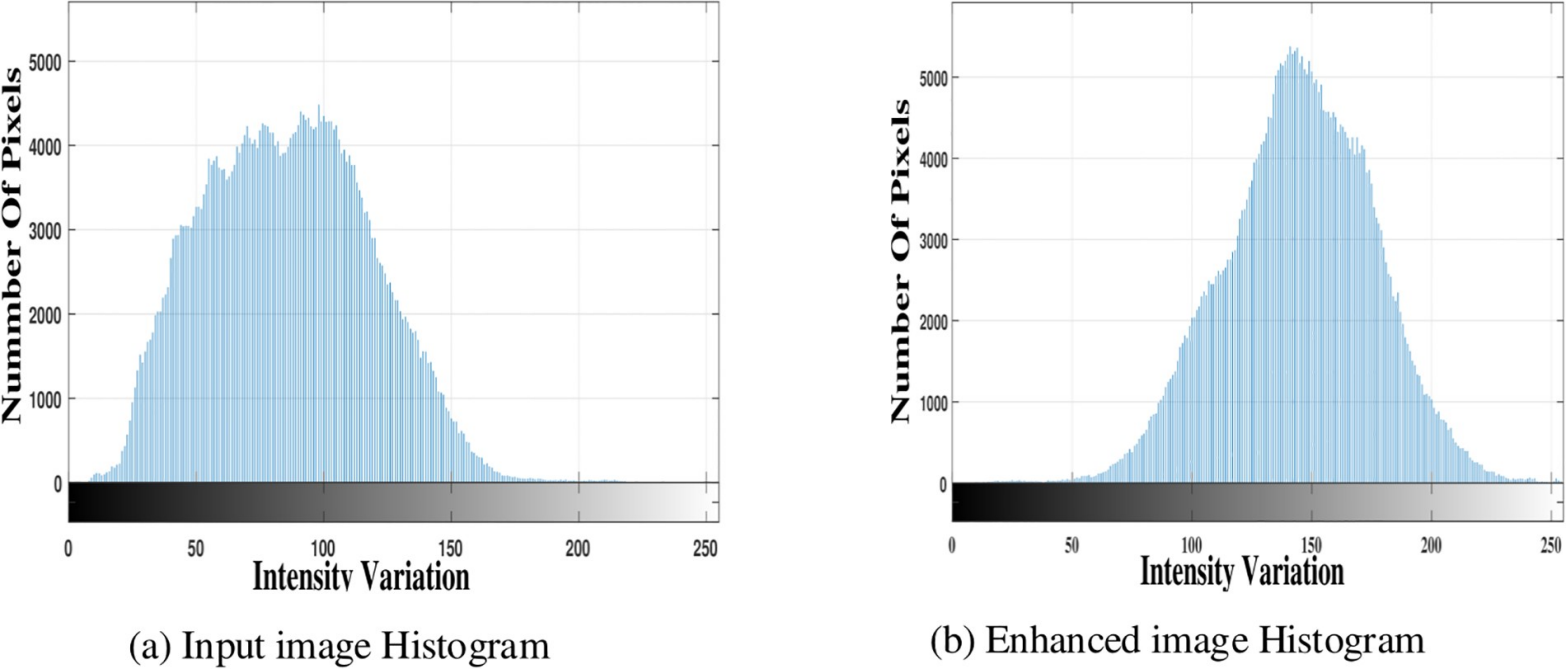
Pixels intensity histograms.

Fig 4 displays the noisy and noise free image. The noise has been removed using DFB. DFB is helpful in enhancing the blur/noisy image by extracting the directional information from the blur part of the image. Fig 5 shows pixels intensity histograms of degraded input image and enhanced image.

### 2.3 Image Segmentation

**Fig 6:**
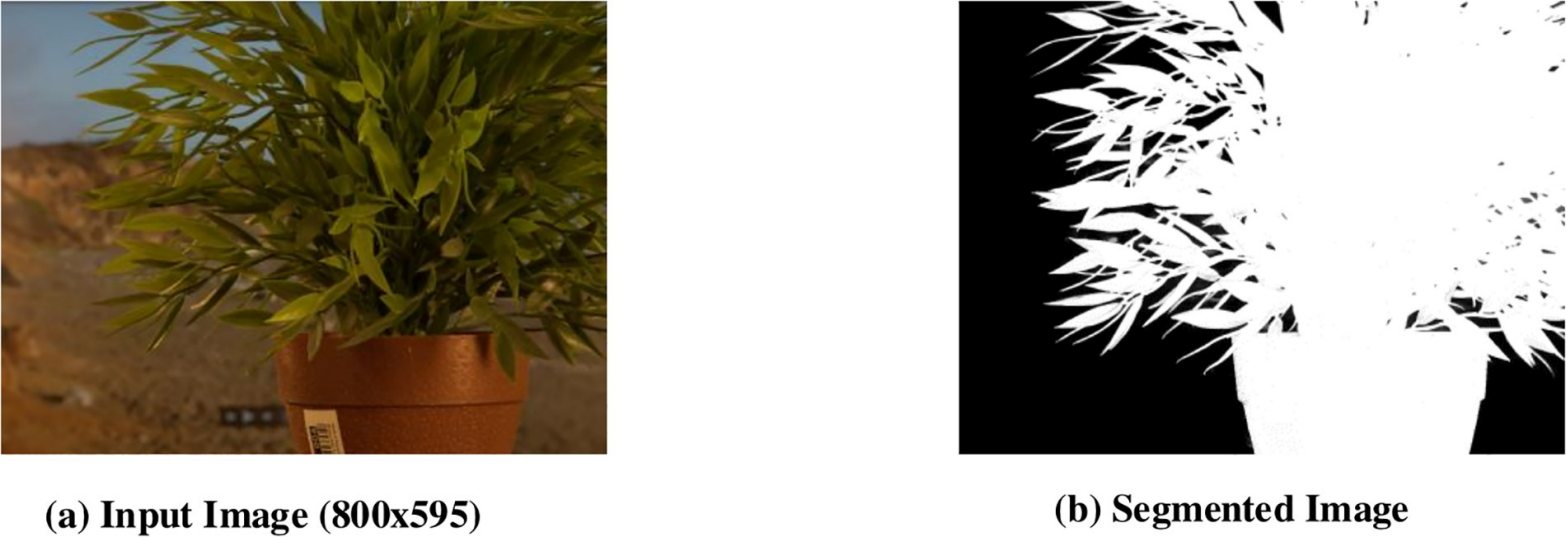
Image Segmentation.

After enhancement of an image by DFB, the input image as shown in Fig 6, is segmented into its foreground image and background image by providing the ground truth of the input image. The foreground image consists of high intensity pixels information whereas background consists of low intensity pixels. It can be seen in Fig 6, the foreground is bright (noise free/blur free) so it will appear ablaze in gray shades of the depth map image. The Background region has intensity variation in the enhanced input image. Pixels of similar intensity should have similar depth value in the depth map. In order to do so, K-mean classify algorithm has been used to group similar intensity pixels as well as count the total number of Pixels contained in each group.

Depth map image is expressed in grayscale where noise free or the enhanced region of the input image is indicated as a bright region and the blur/noisy region is shown as a dark region. In [26], image profiles are generated by finding the vanishing points using Hough transform. The vanishing points in [26] have been considered the farthest region in the input image and accepted to be the dark region in depth map. whereas, the closest points to the viewing position are considered the bright region in depth map. In the DFB-DIBR, the image profile/depth hypothesis is generated without using the vanishing points of Hough transform. By avoiding Hough transform, the efficiency of the proposed algorithm is measured promising in the term of computational complexity. The enhanced region of the input image appears bright in the depth map image and the blur/noisy region appears dark because the farthest points usually become blur/noisy while taking images. The image profile/Depth hypothesis is determined using Euclidean distance formula and the relative height depth cue. The image profile of nth regions can be determined by Eq 8

$$I{}_{E}^{n}\left(x,y\right)=\sqrt{{\left(x-x{}_{v}\right)}^{2}+{\left(y-y{}_{v}\right)}^{2}}/max\left(I{}_{E}^{n}\right)$$ (8)

Where $I{}_{E}^{n}\left(x,y\right)$ is the image profile *x*_*v*_ and *y*_*v*_ are the generalized points and their values are assumed to be 1. Natural scene images are composed of sky and ground. The sky is the upper part of the image and considered the farthest point, therefore that part appeared dark in depth map. Whereas, the ground is assumed to be the nearest part and accepted to be the bright region in the depth map. Therefore, relative height depth cue image profile *I*_*R*_(*x*, *y*) is determined by y coordinates only. The equation of *I*_*R*_ is shown as Eq 9

$$I{}_{R}\left(x,y\right)=\left(I-y\right)/I$$ (9)

Where I is the height of the input image. This image profile/depth hypothesis presents that variation in gray-scale will occur along y-axis only. Our proposed method uses both 8 and 9 hypothesis separately to determine the depth hypothesis for the depth map generation. The equation of *I*_*f*_ (*x*, *y*) can be obtained by combining Eqs 8 and 9 hypothesis i.e.

$$I{}_{f}\left(x,y\right)=I{}_{E}^{n}\left(x,y\right)+I{}_{R}\left(x,y\right)$$ (10)

**Fig 7:**
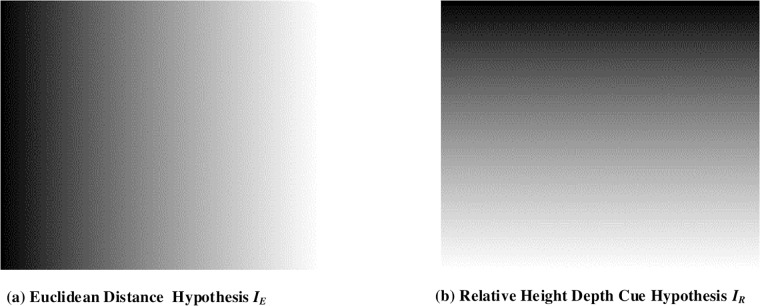
Image profile/depth hypothesis determination.

Fig 7 shows examples of the image profiles, generated by Euclidean distance formula and relative height depth cue.

**Fig 8:**
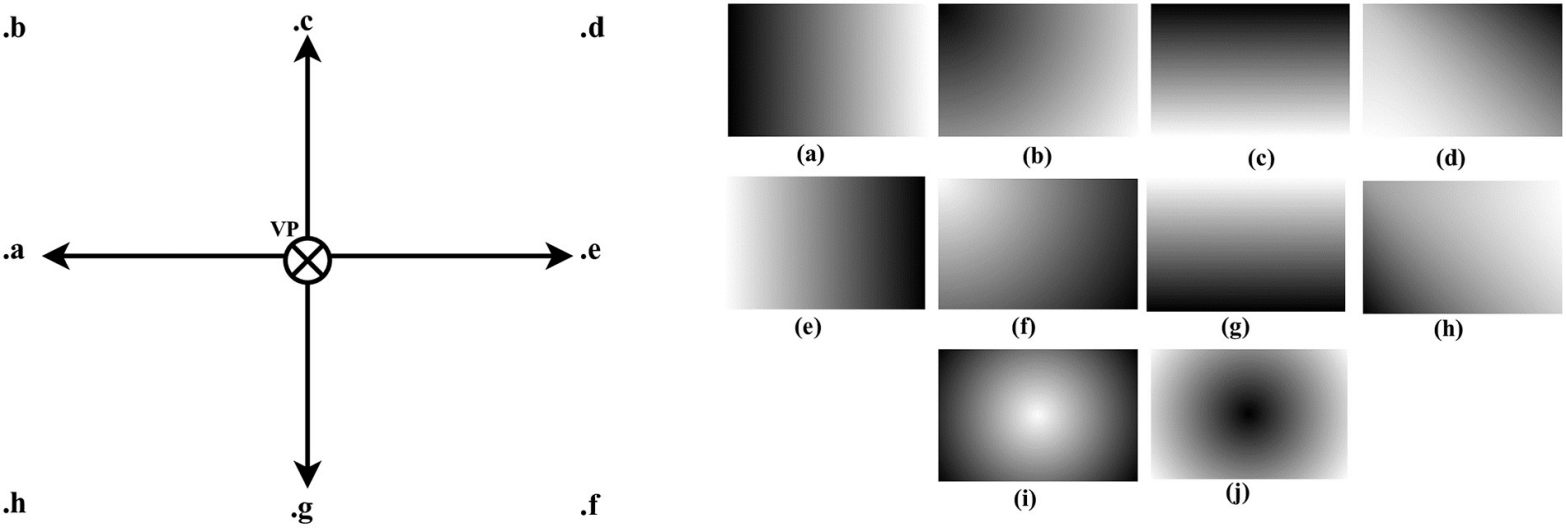
Image profiles/hypothesis generation base on vp (.a, .b, .c, .d, .e, .f, .g, .h, .i, .j).

Fig 8 shows some examples of the image profiles with respect to the location of the viewing point (VP). High intensity regions in the input image will appear bright in image profile and as the intensity decrease of the regions in an input image, those regions will appear dark in image profile. In Fig 8, regions close to the (VP) appeared bright in image profiles because close points to the (VP) have high intensity information and it is the blur/noise free region. As the regions getting far from (VP), image information will become blur/noisy gradually which introduce dark shades in image profile. All image profiles in Fig 6 are generated by Euclidean distance except Fig 8(c) and 8(g) which are generated by the relative height depth cue. The concept behind Fig 8(i) is to focus the farthest middle regions in the input image and blurred the nearest region so the farthest region is bright in image profile and the nearest region is darker. For example, we usually watch scenes in a movie where the farthest object is focused and the nearest region appeared blur. The inverse phenomenon of Fig 8(i) is shown in Fig 8(j). After generating image profiles, depth map *D*_*map*_ is generated. In order to generate depth map *D*_*map*_, image profiles *I*_*pro*_ values are assigned to each segmented regions S in the previous step. The *D*_*map*_ value at any given point *D*_*map*_(*x*, *y*) is computed by average *I*_*pro*_ and the average depth value of the segmented regions S(x,y), *I*_*pro*_. The average value of the segmented region S(x,y) and Eq 11 *I*_*spro*_ can be computed as

$$I{}_{Spro}=\frac{1}{T{}_{\left(n(x,y)\right)}}\sum I{}_{pro}\left(p,q\right)$$ (11)

where *T*_(*n*(*x*,*y*))_ shows the total number of pixels in a segmented S(x, y) region. We assumed that regions of similar intensity have the same depth value in *D*_*map*_ but this concept does not hold for the large region. As the larger region contains an excessive number of pixels. Therefore we check the number of pixels in each segmented region S(x,y) by

$$D{}_{map}=\{\begin{array}{c} I{}_{spro}\;if\;T{}_{n(x,y)}<T{}_{th}\\ \frac{1}{2}\left(I{}_{pro}+I{}_{spro}\right)\;otherwise \end{array}$$ (12)

Where *T*_*th*_ is the threshold, used to check the size of the region by counting the number of pixels in a region. If the region has less number of pixels which possess small depth variation then *D*_*map*_ is calculated by *I*_*spro*_. Otherwise, *D*_*map*_ is calculated by averaging both image profiles *I*_*pro*_ and *I*_*spro*_.

### 2.4 Depth map Refinement

**Fig 9:**
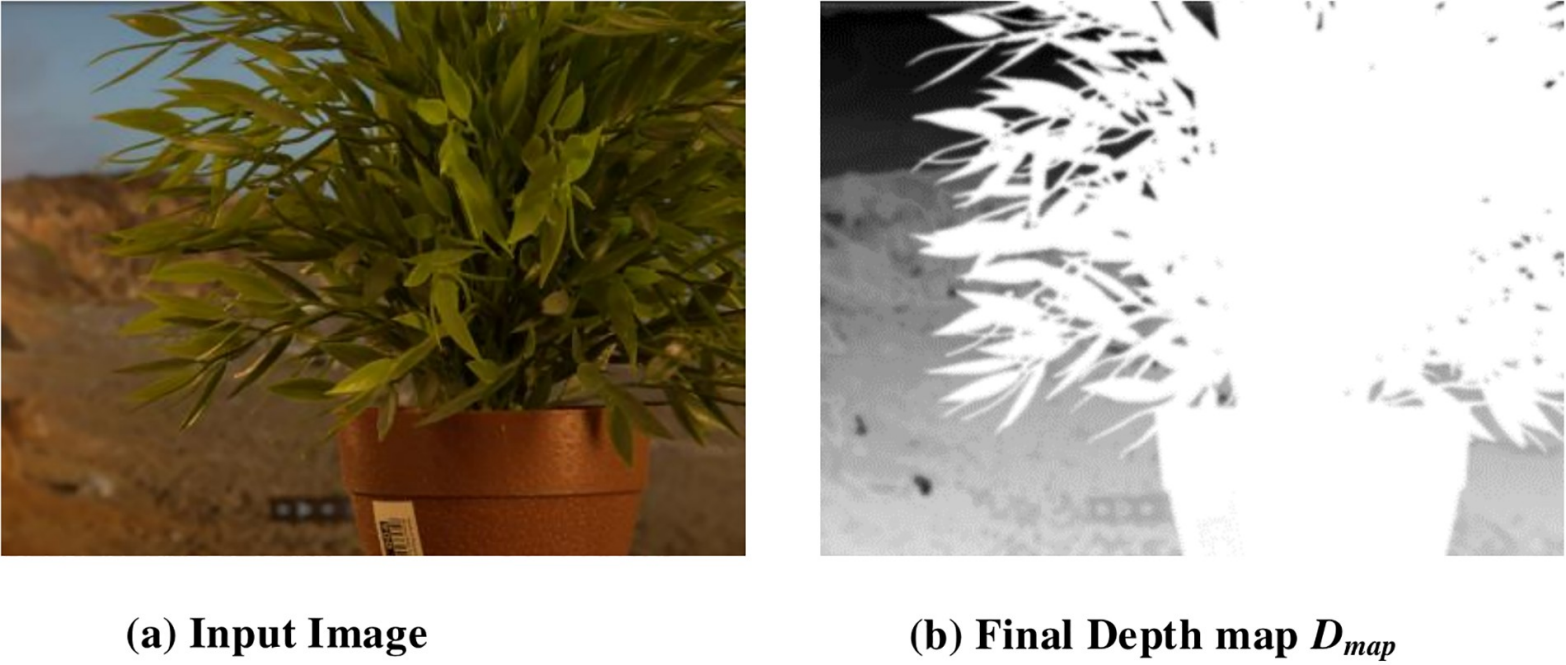
Refined depth map *D*_*map*_.

In the *D*_*map*_, pixels of segmented regions differ across different depth value than those of the neighboring pixels though they must have the same depth value that relates to the same region in the input image. If a region in the input image with the same depth is divided into several regions with different depth, it creates unnatural artifacts. To avoid such unnatural artifacts, the adaptive bilateral filter is applied to produce a refined depth map. The input image and its related depth map *D*_*map*_ are shown in Fig 9.

### 2.5 Depth Image based Rendering

Depth Image Based Rendering is the procedure to generate the synthesized 3D virtual view of a scene by combining the reference input image and the depth map. In [36], the DIBR is based on two different synthesized images i.e. the virtual left eye and the right eye camera parameters which are obtained by using 3D warping Eqs 13 and 14

$$\left(X{}_{r},y\right)=X{}_{c}-\frac{t{}_{x}}{2}*\frac{f}{d}$$ (13)

$$\left(X{}_{l},y\right)=X{}_{c}+\frac{t{}_{x}}{2}*\frac{f}{d}$$ (14)

where (*X*_*r*_), (*X*_*l*_) are the right and left image pixels positions, *X*_*c*_ is the center of the image, *t*_*x*_ is the baseline or distance between two lenses of a camera, f is the focal length while d being the depth map of the respective image. Occlusion may occur after the translation of the input image into synthesized left and right images. To handle the occluded area, the neighborhood pixels textures are averaged to fill the newly exposed area in synthesized images.

### 2.6 Handling occlusion

Input images are translated to stereoscopic (left and right) images using DIBR procedure. Occlusion occurred whenever images are translated which leaves holes in translated images. The occluded area may be visible in virtual views which can be filled by using the background pixels using the following Eq 15

$$I\left(x,y\right)=\frac{\sum_{n=1}^{w}B{}_{g}\left(i,y\right)}{w}$$ (15)

Where *I*(*x*, *y*) represents the occluded point location in coordinate (*x*, *y*), *B*_*g*_(*i*, *y*) is the background pixel in coordinate (*i*, *y*) only horizontal pixels are computed to fill the holes and *w* stands for window size. After holes filling, a median filter is used to smooth the filled area.

## 3 Experimental results and discussion

**Fig 10:**
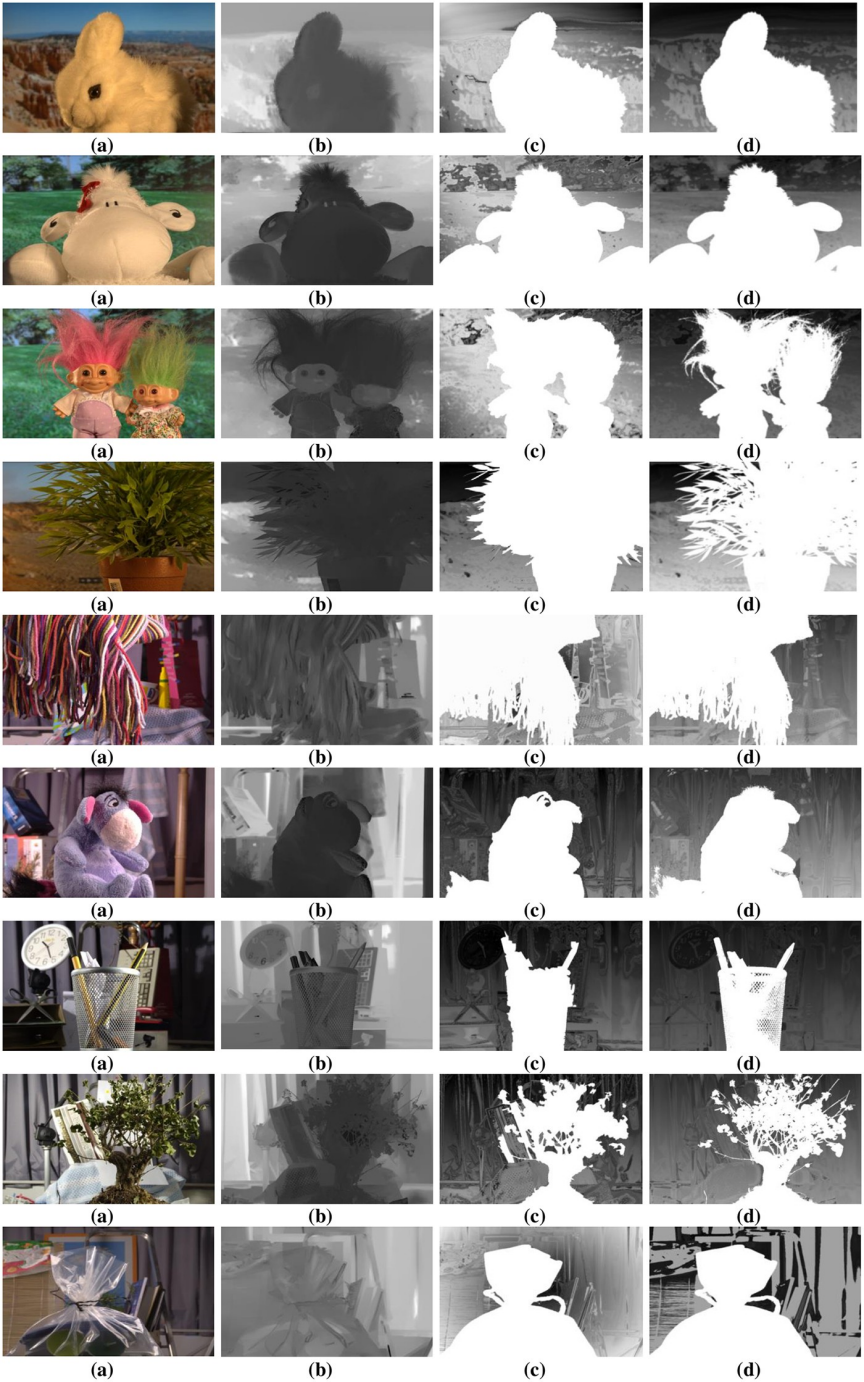
Depth map results of dataset [37]. (**a**) Input Images (**b**) Depthmap produced by Zhuo.et al. [12] (**c**) Depthmap produced by Yang.et.al. [26] and (**d**) Depth-map generated by DFB-DIBR.

**Fig 11:**
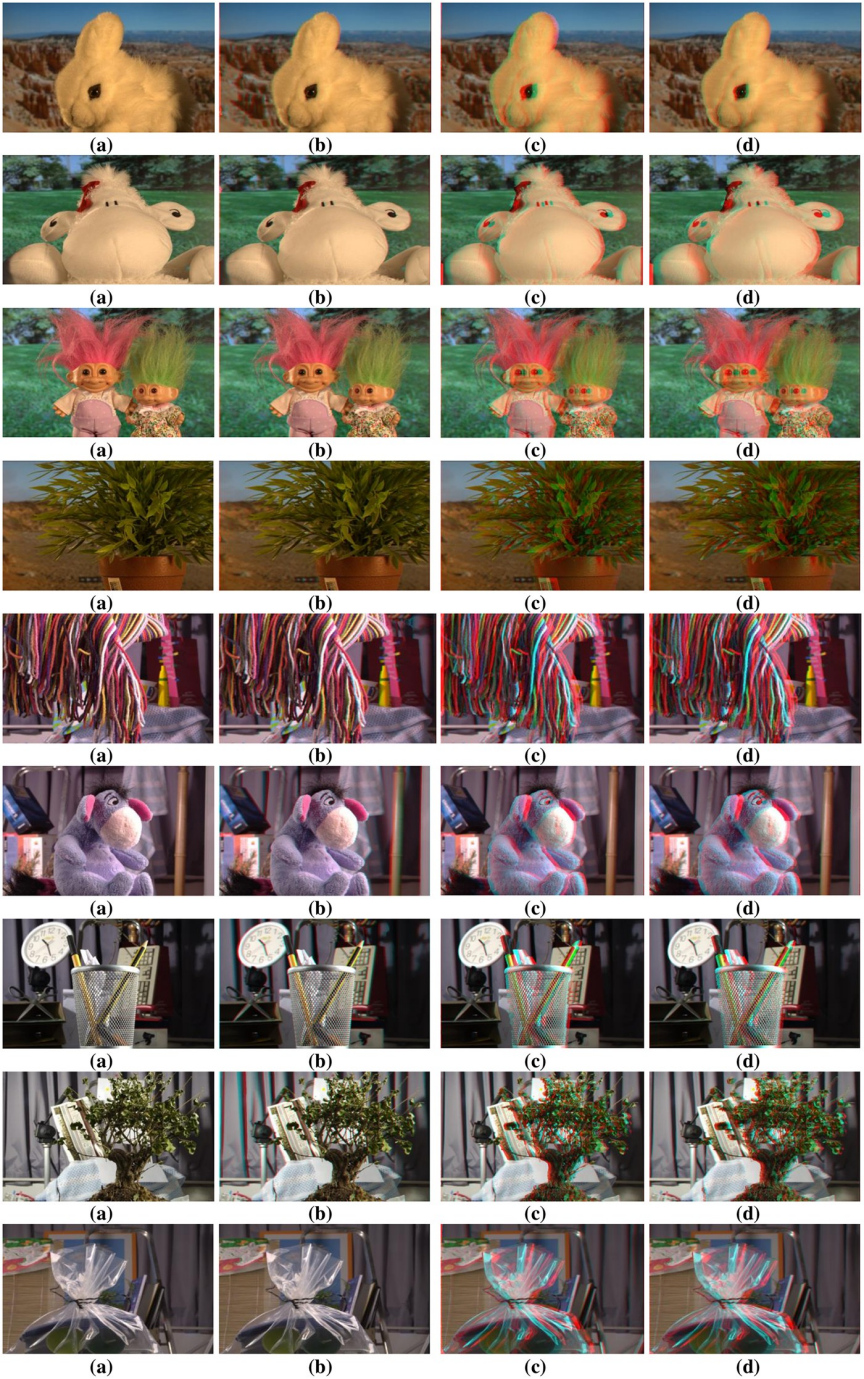
Anaglyph results of dataset [37]. (**a**) Input Images (**b**) Anaglyph produced by Zhuo.et al. [12] (**c**) Anaglyph produced by Yang.et.al. [26] and (**d**) Anaglyph generated by DFB-DIBR.

To authenticate the predominance of the DFB-DIBR, numbers of degraded images datasets [37–39] have been tested. In the dataset [37] tested images are Rabbit(800x490), Bear(800x618), Troll(800x563), plant2(800x595), Threads(800x608), Donkey(800x543), Glass(800x532), Plant(800x604) and Plastic bag(800x662). The Data set [37] consists of two types of degraded images. First type of degraded images are photographed by placing the object in front of the monitor seeming natural blur/noisy images. Second type of degraded images are taken in real natural view. The experiments are conducted using a System with Intel Core i7-3632QM CPU(2.20 GHz) having 8GB of RAM. To assess the results of the DFB-DIBR and state of the art methods [12] and [26], the depth map and corresponding anaglyph images have been shown in Figs 10 and 11 respectively. The depth map results of fabricated blur/noisy images, generated by [12] and [26] cannot distinguish the architecture very well. In test sequence “Glass” the texture information of digits written inside the clock is missing in the depth map of [12] and [26]. Whereas such texture information is very much clear in the depth map of the DFB-DIBR due to enhancement of the background data. The relationship between depth values of different intensity pixels are ignored in [12], especially the depth values of different objects are same in the tested image “Threads” which in fact, contain different intensity values and should have assigned different depth values. The proposed system clearly differentiates the high and low-intensity pixels and assigned respective depth values accordingly. The edges information of sky in the test sequence “Rabbit” is missing in the depth map of [12] and [26]. The depth map results of blur/noisy images taken in natural view of the purposed system are far superior than the depth map results of Refs [12, 26]. The depth map of the test sequence “plant2” is properly bedded. In other words, the global depth gradient is maintained by the proposed system whereas such property is ignored by [12] and [26]. To show the performance dominance of the proposed system over conventional algorithms [12] and [26], some full-reference image quality evaluation parameters are used such as Universal image quality index (UQI) [44], Structural Similarity Index (SSIM) [45] and Peak Signal to Noise Ratio (PSNR) [46].

**Fig 12:**
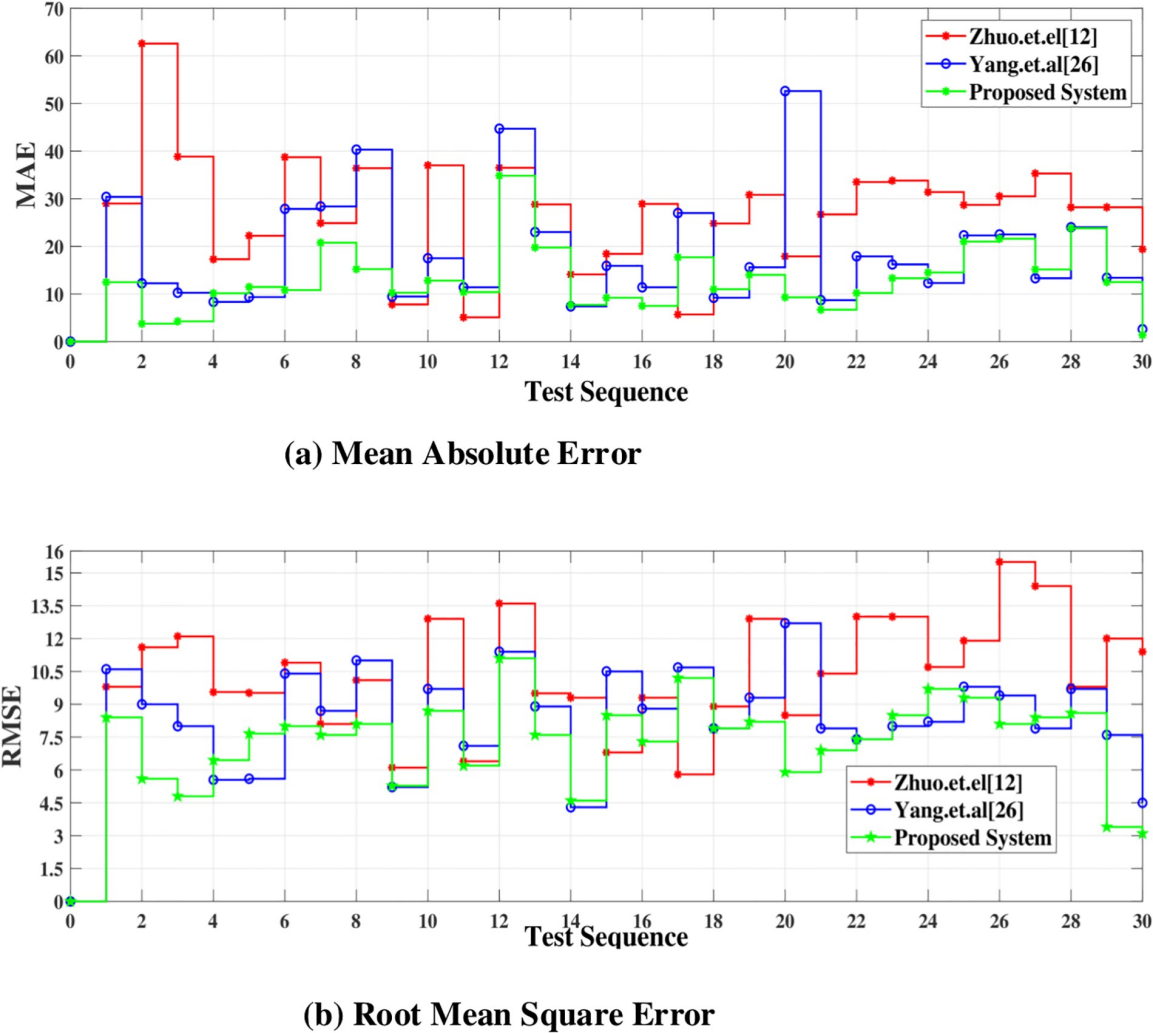
MAE and RMSE of the dataset [37].

The statistical evaluation validate that the proposed system, compared with [12] and [26], can estimate the efficient depth map. In PSNR, SSIM and UQI higher values are considered better. Compare to [12] and [26], the values of the SSIM of the proposed system are better than [12] and [26]. At the same time, the PSNR value of proposed system compare to [12] and [26], increases by 1.399 and 1.024 dB averagely. As to the UQI, the average value of UQI of the proposed system is higher than [26] about 0.106. whereas, it is higher than [12] about 0.054 averagely. The bold values in the Table 1 are considered better. In PSNR, SSIM and UQI higher values are better. Mean Absolute Error(MAE) and Root Mean Square Error(RMSE) have been calculated of depth map obtained by Refs [12, 26] and the proposed system. MAE and RMSE are shown in Fig 12. It is clear from the Fig 12 that error in depth map images generated by the proposed system is very low as compare to [12] and [26].

**Table 1:** Comparison parameters PSNR, SSIM and UQI using dataset [37].

Image	Zhuo et.al [12]			Yang et.al [26]			Proposed System		
	PSNR	SSIM	UQI	PSNR	SSIM	UQI	PSNR	SSIM	UQI
1	8.754	0.566	0.632	9.403	0.486	**0.686**	**19.226**	**0.672**	0.630
2	7.119	0.442	0.456	10.672	0.710	0.757	**21.734**	**0.789**	**0.850**
3	11.761	0.436	0.690	8.183	0.487	0.682	**16.796**	**0.489**	**0.691**
4	15.773	0.535	0.853	6.985	0.272	0.325	**17.563**	**0.552**	**0.753**
5	11.215	0.272	0.658	9.492	**0.325**	0.443	**15.403**	0.314	**0.692**
6	7.666	0.309	0.514	8.359	0.424	0.599	**16.711**	**0.481**	**0.666**
7	7.055	0.171	0.448	8.052	0.287	0.453	**18.108**	**0.359**	**0.479**
8	9.456	**0.338**	**0.667**	6.294	0.260	0.551	**13.475**	0.285	0.604
9	9.625	0.489	0.641	13.891	0.435	0.582	**17.021**	**0.573**	**0.786**

**Fig 13:**
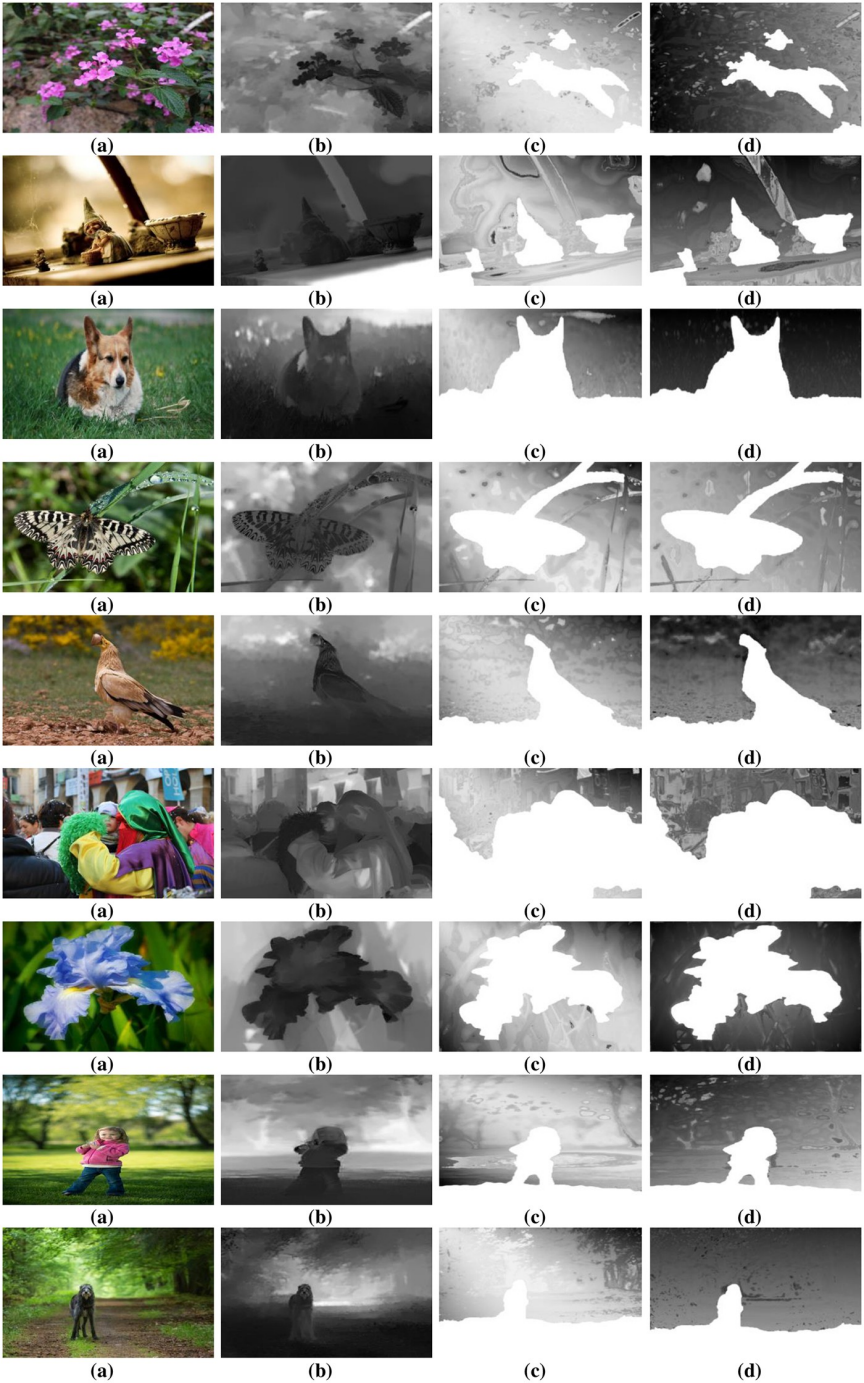
Depth map results of dataset [38]. (**a**) Input Images (**b**) Depthmap produced by Zhuo.et al. [12] (**c**) Depthmap produced by Yang.et.al. [26] and (**d**) Depth-map generated by DFB-DIBR.

**Fig 14:**
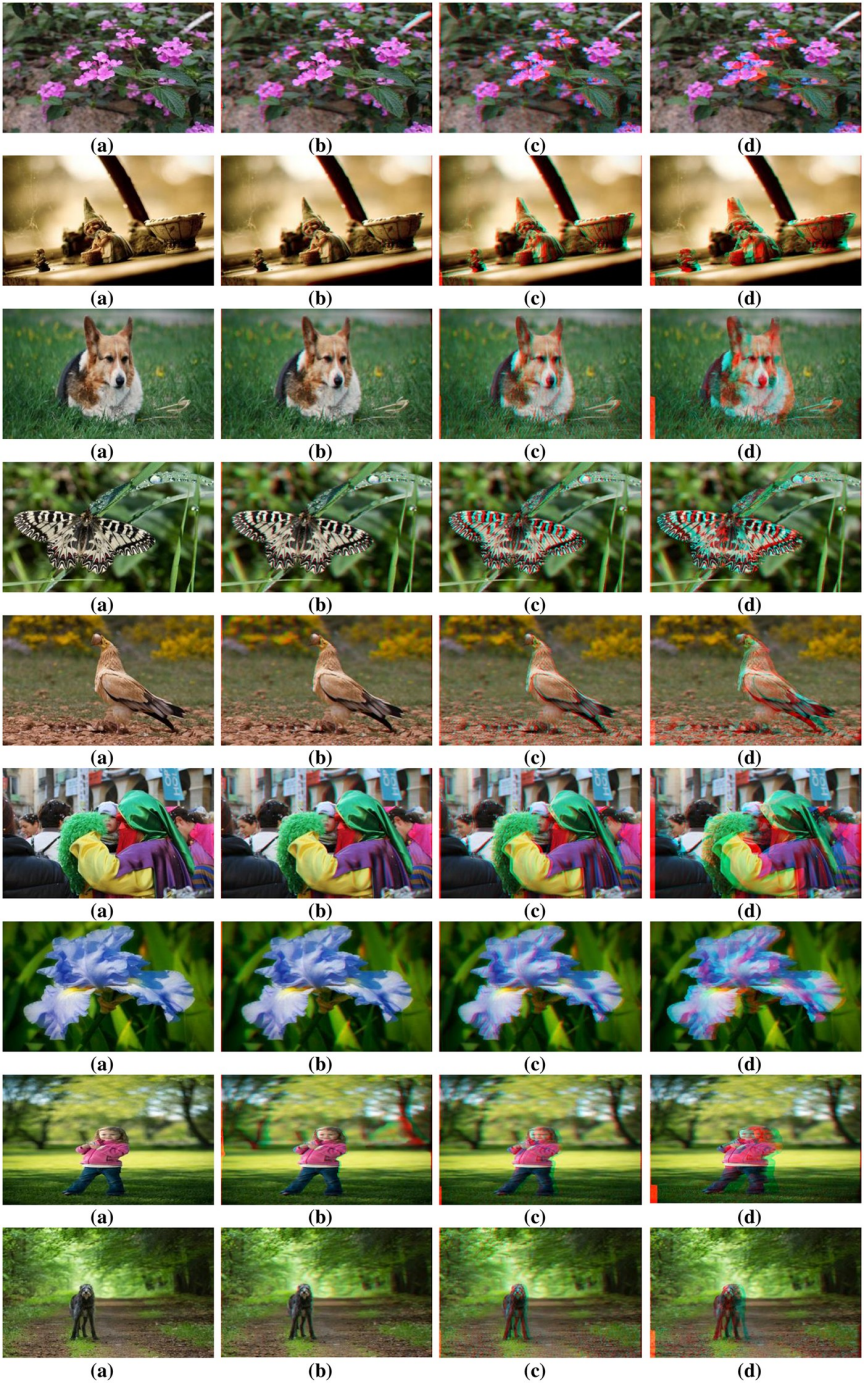
Anaglyph results of dataset [38]. (**a**) Input Images (**b**) Anaglyph produced by Zhuo.et al. [12] (**c**) Anaglyph produced by Yang.et.al. [26] and (**d**) Anaglyph generated by DFB-DIBR.

**Fig 15:**
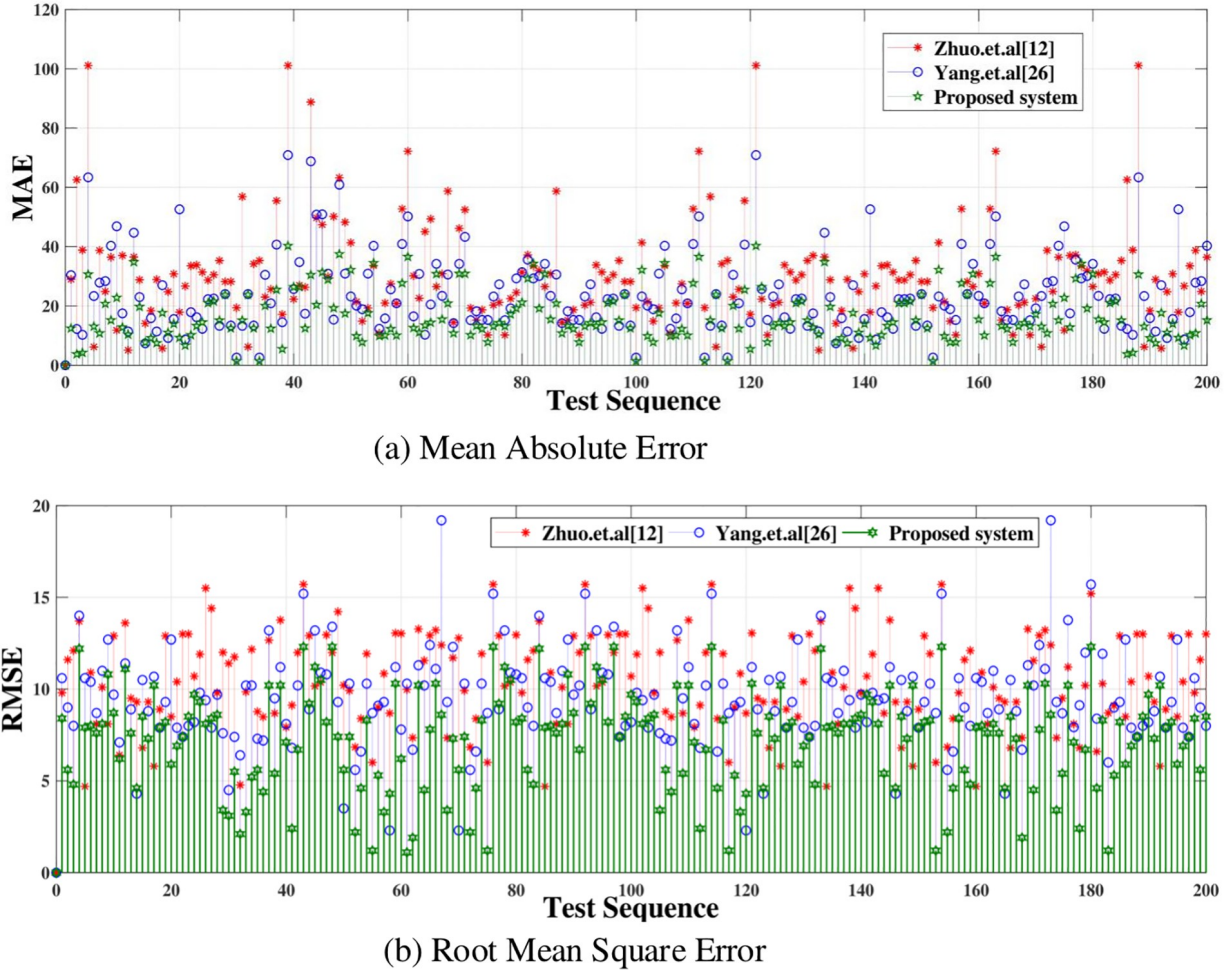
MAE and RMSE of the dataset [38].

The proposed system has been tested using another dataset [38]. The dataset includes more than 500 degraded images of different types i.e. indoor, outdoor, people, building, car etc. The PSNR, SSIM and UQI have been calculated of the depth map generated by proposed, [12] and [26]. Their values are displayed in Table 2. The depth map and anaglyph results of dataset [38] are shown in Figs 13 and 14 respectively. The MAE and RMSE of the depth map generated by proposed system, [12] and [26] is calculated using dataset [38]. The MAE and RMSE are shown in Fig 15 respectively.

**Table 2:** Comparison parameters PSNR, SSIM and UQI using dataset [38].

Image	Zhuo et.al [12]			Yang et.al [26]			Proposed System		
	PSNR	SSIM	UQI	PSNR	SSIM	UQI	PSNR	SSIM	UQI
1	11.292	0.302	0.671	5.823	0.282	0.501	**18.744**	**0.597**	**0.685**
2	6.352	0.406	0.423	6.823	0,427	**0.638**	**15.236**	**0.527**	0.403
3	11.393	**0.493**	**0.592**	6.017	0.345	0.587	**14.297**	0.366	0.413
4	9.497	0.244	0.577	5.864	0.253	0.523	**17.087**	**0.428**	**0.626**
5	10.824	0.465	0.662	7.233	0.506	0.638	**17.089**	**0.427**	0.578
6	10.953	0.392	**0.668**	4.865	0.337	0.538	**14.328**	**0.45i**	0.553
7	7.665	0.363	0.405	6.267	0.473	0.286	**19.217**	**0.576**	**0.668**
8	10.955	**0.503**	**0.795**	6.967	0.507	0.614	**13.407**	0.357	0.638
9	11.775	0.393	0.587	6.337	0.413	0.526	**16.703**	**0.435**	**0.668**

The proposed system has been tested using enhanced images’ dataset [39]. Enhanced images do not require dehazing and DFB based enhancement. To check the performance of the proposed system, the depth map results are compared with the depth map results generated by state of the art algorithm [8]. Full reference 2D image evaluation parameters PSNR,SSIM and VIF [47] have been used to evaluate the quality of the depth map. The results of the depth map are shown in Table 3. It is clear from the Table 3 that the PSNR and VIF values of the proposed system are dominant over the [8] i.e. 6.4 and 0.081 averagely. At the same time, the average SSIM values of the [8] are higher than the proposed system about 0.06.

**Table 3:** Comparison parameters PSNR, SSIM and VIF using dataset [39].

Image	Yang et.al [8]			Proposed System		
	PSNR	SSIM	VIF	PSNR	SSIM	VIF
Swords	12.36	**0.65**	0.04	**19.25**	0.53	**0.08**
Umbrella	13.24	0.70	0.05	**20.15**	**0.75**	**0.21**
Aloe	15.48	0.57	0.10	**18.30**	**0.58**	**0.21**
Ballet	12.44	**0.70**	0.12	**24.34**	0.64	**0.19**
Road	10.45	**0.63**	0,09	**13.92**	0.45	**0.12**

### 3.1 3D view evaluation

**Fig 16:**
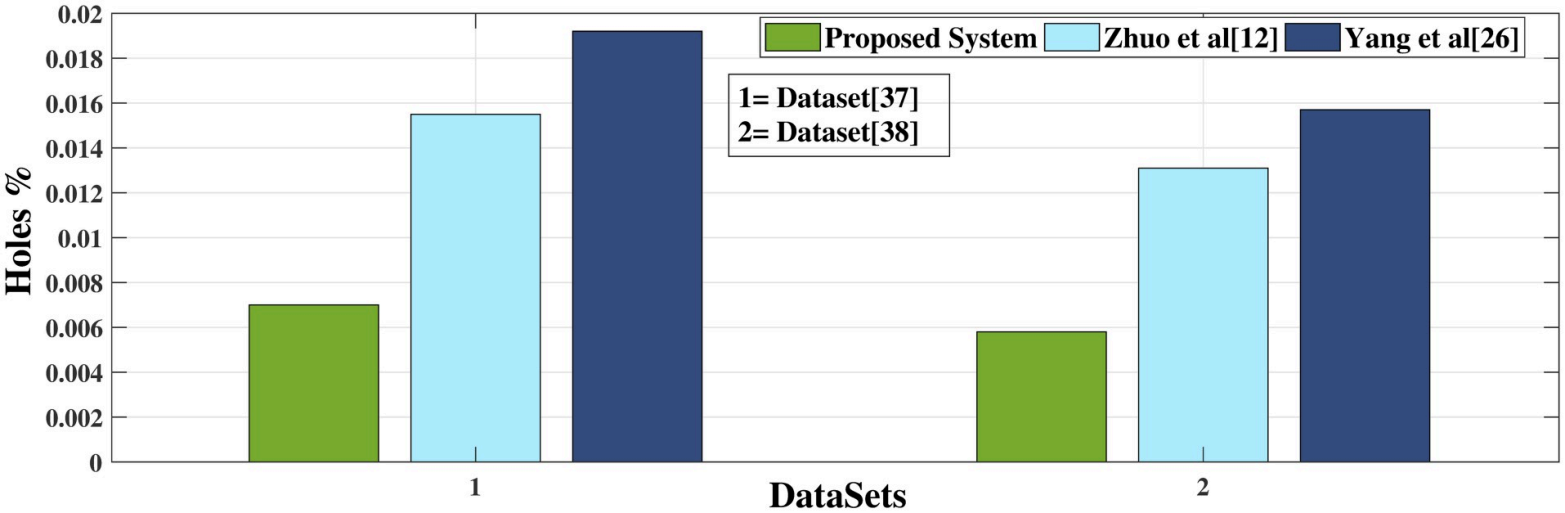
Holes percentage of occluded area.

To evaluate the 3D results of proposed system, the percentage of holes in the occluded area has calculated in translated images. The value of the percentage is quite minimal almost 0.006% and 0.007% averagely. This minimum value of the percentage shows that the proposed system generates quality depth map which ultimately creates a presentable 3D scene. Data about holes in the occluded area has been calculated using block size 8x8 and 16x16 which can be seen in the Tables 4 and 5. In Tables 4 and 5 LI refers to Left Image and RI refers to Right Image. The hole percentage of the tested datasets [37] and [38] is displayed in Fig 16.

**Table 4:** Holes percentage using block size 8x8 and block size 16x16 tested dataset [37].

Holes Percentage Using Block size 8x8							Holes Percentage Using Block size 16x16					
Image	Zhuo et al. [12]		Yang et al. [26]		Proposed Method		Zhuo et al. [12]		Yang et al. [26]		Proposed Method	
	LI	RI	RI	RI	LI	RI	LI	RI	LI	RI	LI	RI
1	0.011	0.011	0.023	0.023	0.007	0.007	0.011	0.011	0.023	0.023	0.005	0.005
2	0.010	0.010	0.025	0.025	0.008	0.008	0.010	0.010	0.025	0.025	0.005	0.005
3	0.010	0.010	0.030	0.031	0.019	0.019	0.010	0.017	0.030	0.031	0.009	0.009
4	0.010	0.010	0.025	0.025	0.002	0.002	0.011	0.011	0.027	0.027	0.003	0.003
5	0.015	0.015	0.058	0.058	0.001	0.001	0.016	0.016	0.059	0.060	0.002	0.002
6	0.014	0.014	0.017	0.017	0.007	0.007	0.014	0.014	0.017	0.017	0.007	0.007
7	0.012	0.012	0.018	0.018	0.009	0.009	0.012	0.012	0.018	0.018	0.009	0.009
8	0.015	0.015	0.050	0.049	0.022	0.022	0.016	0.015	0.050	0.049	0.022	0.022
9	0.010	0.010	0.034	0.034	0.005	0.005	0.015	0.015	0.038	0.038	0.006	0.006

**Table 5:** Holes percentage using block size 8x8 and block size 16x16 tested dataset [38].

Holes Percentage Using Block size 8x8							Holes Percentage Using Block size 16x16					
Image	Zhuo et al. [12]		Yang et al. [26]		Proposed Method		Zhuo et al. [12]		Yang et al. [26]		Proposed Method	
	LI	RI	RI	RI	LI	RI	LI	RI	LI	RI	LI	RI
1	0.010	0.012	0.018	0.018	0.009	0.009	0.010	0.012	0.018	0.018	0.009	0.009
2	0.012	0.012	0.034	0.035	0.010	0.010	0.012	0.012	0.034	0.035	0.010	0.010
3	0.011	0.011	0.011	0.011	0.005	0.005	0.011	0.011	0.011	0.011	0.005	0.005
4	0.023	0.023	0.014	0.014	0.010	0.010	0.015	0.015	0.014	0.014	0.010	0.010
5	0.010	0.010	0.012	0.012	0.006	0.007	0.010	0.010	0.012	0.012	0.006	0.007
6	0.015	0.015	0.014	0.014	0.010	0.010	0.015	0.015	0.014	0.014	0.010	0.010
7	0.013	0.013	0.013	0.013	0.005	0.005	0.013	0.013	0.013	0.013	0.005	0.005
8	0.026	0.026	0.018	0.018	0.009	0.009	0.026	0.026	0.018	0.018	0.009	0.009
9	0.009	0.009	0.016	0.016	0.005	0.005	0.009	0.009	0.016	0.016	0.005	0.005

## 4 Conclusion

In this paper, we proposed a novel approach to convert a 2D blur/noisy image to 3D view. The image is dehazed first. Then the noisy image is enhanced using DFB. The enhanced image is segmented into background and foreground in the next stage. The foreground is the enhanced part of the image and the background part has intensity variation. The similar intensities are grouped using k-mean algorithm. After grouping similar intensities, image profile/depth hypothesis procedure is applied to generate depth map. The initial depth map is further refined using a bilateral filter to remove some natural artifacts. Moreover, the stereoscopic images are produced using DIBR. Experimental results show the superiority of the proposed novel approach to generate 3D scene from single 2D blur/noisy image. Since the proposed system generates efficient results therefore the future research will focus on using the proposed system as hand crafted feature for the deep learning algorithm.
